# Supplementary material for: ESCRT-0 regulates AMPA receptor currents and Ca2+- dependent signaling
Source: bioRxiv. 2026 Jun 24:2026.06.19.733273. Preprint. [Version 1] doi: 10.64898/2026.06.19.733273 (PMC13320757; doi:10.64898/2026.06.19.733273)
Supplement: Supplement 2 [file NIHPP2026.06.19.733273v1-supplement-2.pdf]

bioRxiv preprint doi: https://doi.org/10.64898/2026.06.19.733273; this version posted June 24, 2026. The copyright holder for this preprint (which was not certified by peer review) is the author/funder, who has granted bioRxiv a license to display the preprint in perpetuity. It is made available under a CC-BY 4.0 International license.

Supplementary Figures

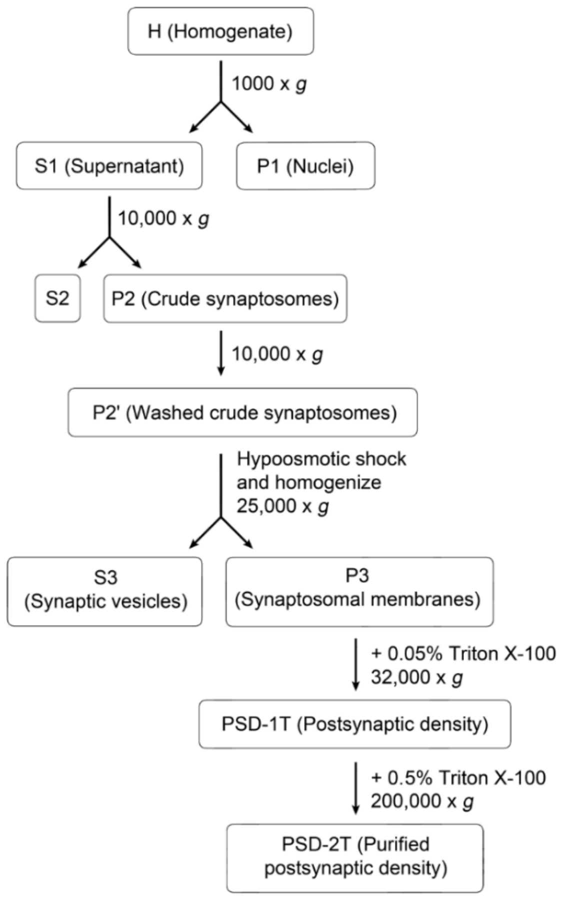

Figure S1. Schematic diagram depicting post-synaptic membrane (PSD2) purification protocol from WT mouse brain (cortex and hippocampus).

bioRxiv preprint doi: https://doi.org/10.64898/2026.06.19.733273; this version posted June 24, 2026. The copyright holder for this preprint (which was not certified by peer review) is the author/funder, who has granted bioRxiv a license to display the preprint in perpetuity. It is made available under a CC-BY 4.0 International license.

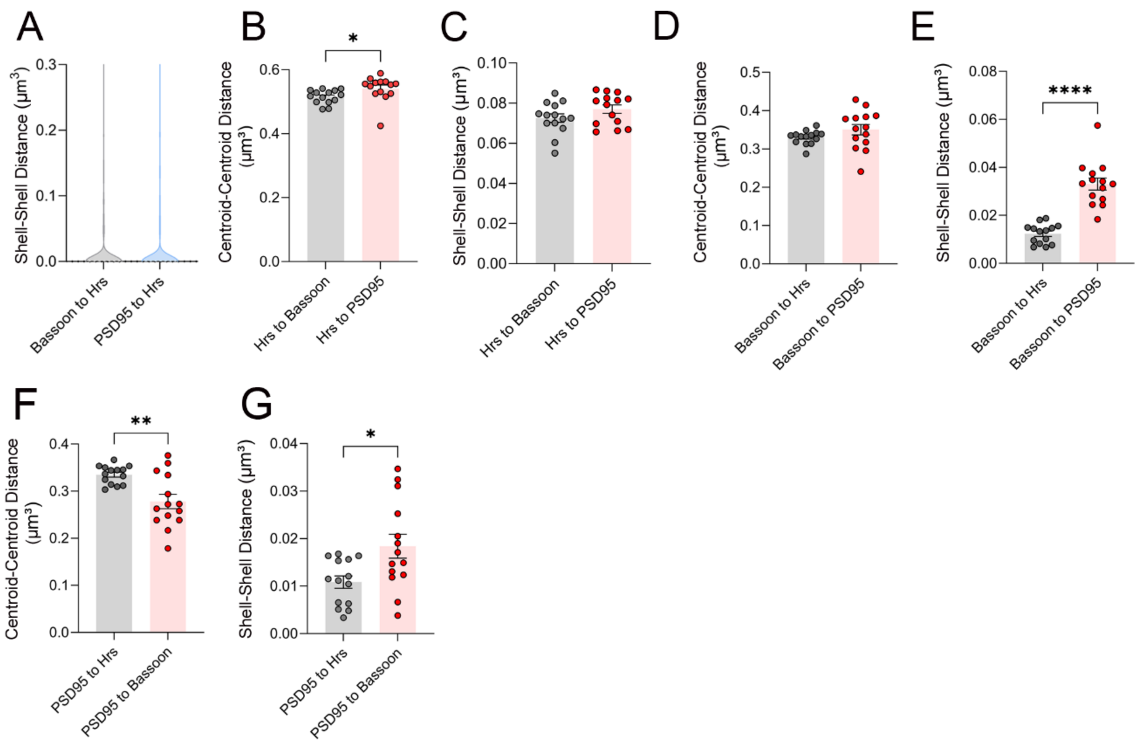

Figure S2. Nanoscale spatial relationship of Hrs with synaptic markers. (A–G) Quantification of nanoscale distances between Hrs and synaptic markers using super-resolution imaging. (A, C, E, G) Shell-to-shell distance measurements represent the minimum edge-to-edge distance between segmented objects. (B, D, F) Centroid-to-centroid measurements represent the distance between object centers. Distances were calculated between Hrs and the presynaptic marker Bassoon and between Hrs and the postsynaptic marker PSD-95. Additional pairwise analyses comparing Bassoon and PSD-95 are shown. Data are presented as mean ± SEM. Statistical significance was determined using two-tailed unpaired t-tests (**p* < 0.05, ***p* < 0.01, ****p* < 0.001).

bioRxiv preprint doi: https://doi.org/10.64898/2026.06.19.733273; this version posted June 24, 2026. The copyright holder for this preprint (which was not certified by peer review) is the author/funder, who has granted bioRxiv a license to display the preprint in perpetuity. It is made available under a CC-BY 4.0 International license.

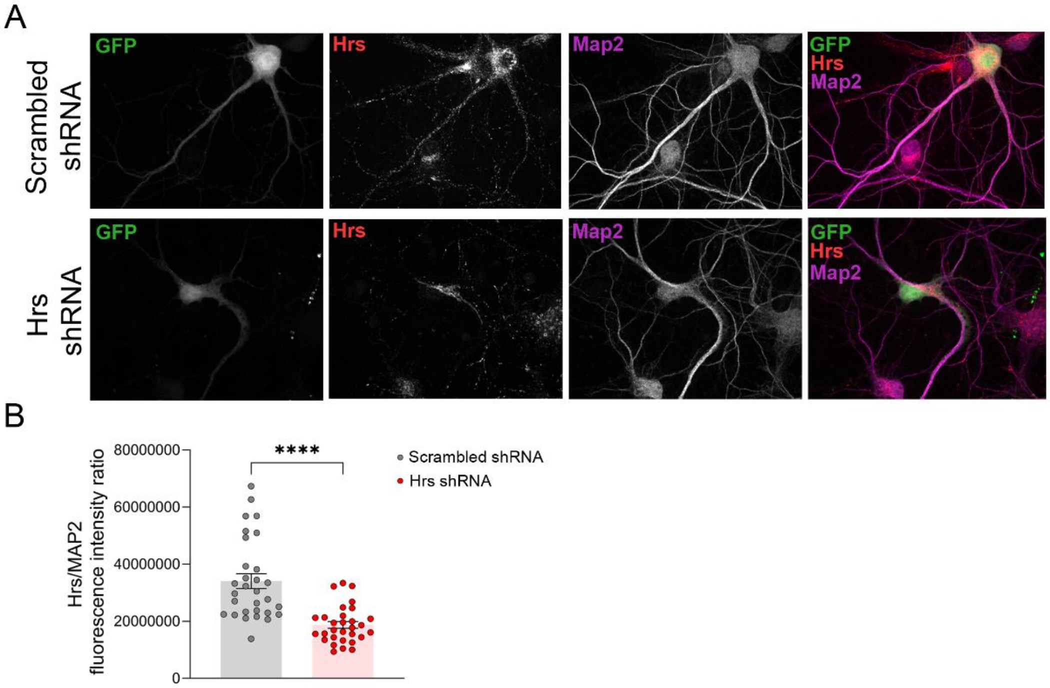

Figure S3. shRNA-mediated knockdown and immunostaining of Hrs in primary hippocampal neurons. Primary hippocampal neurons were transduced with shRNA-expressing lentivirus (Hrs-shRNA or scrambled shRNA) at 5 days in vitro (5 DIV) using 1 µL of virus per coverslip. At 15 DIV, neurons were fixed and immunostained for Hrs, MAP2, and GFP. Hrs/Map2 fluorescence intensity was quantified, revealing a significant reduction in Hrs levels in the Hrs-shRNA–treated neurons compared with control neurons. Unpaired two-tailed *t*-test. **** $p < 0.0001$.

bioRxiv preprint doi: https://doi.org/10.64898/2026.06.19.733273; this version posted June 24, 2026. The copyright holder for this preprint (which was not certified by peer review) is the author/funder, who has granted bioRxiv a license to display the preprint in perpetuity. It is made available under a CC-BY 4.0 International license.

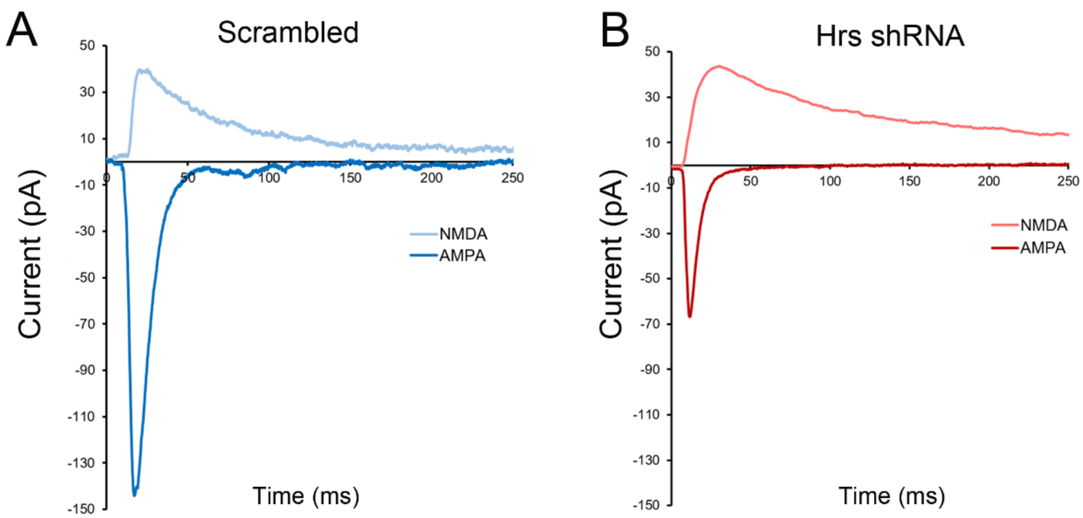

1097 **Figure S4.** Example traces of NMDA and AMPA currents from the same neuron.

bioRxiv preprint doi: https://doi.org/10.64898/2026.06.19.733273; this version posted June 24, 2026. The copyright holder for this preprint (which was not certified by peer review) is the author/funder, who has granted bioRxiv a license to display the preprint in perpetuity. It is made available under a CC-BY 4.0 International license.

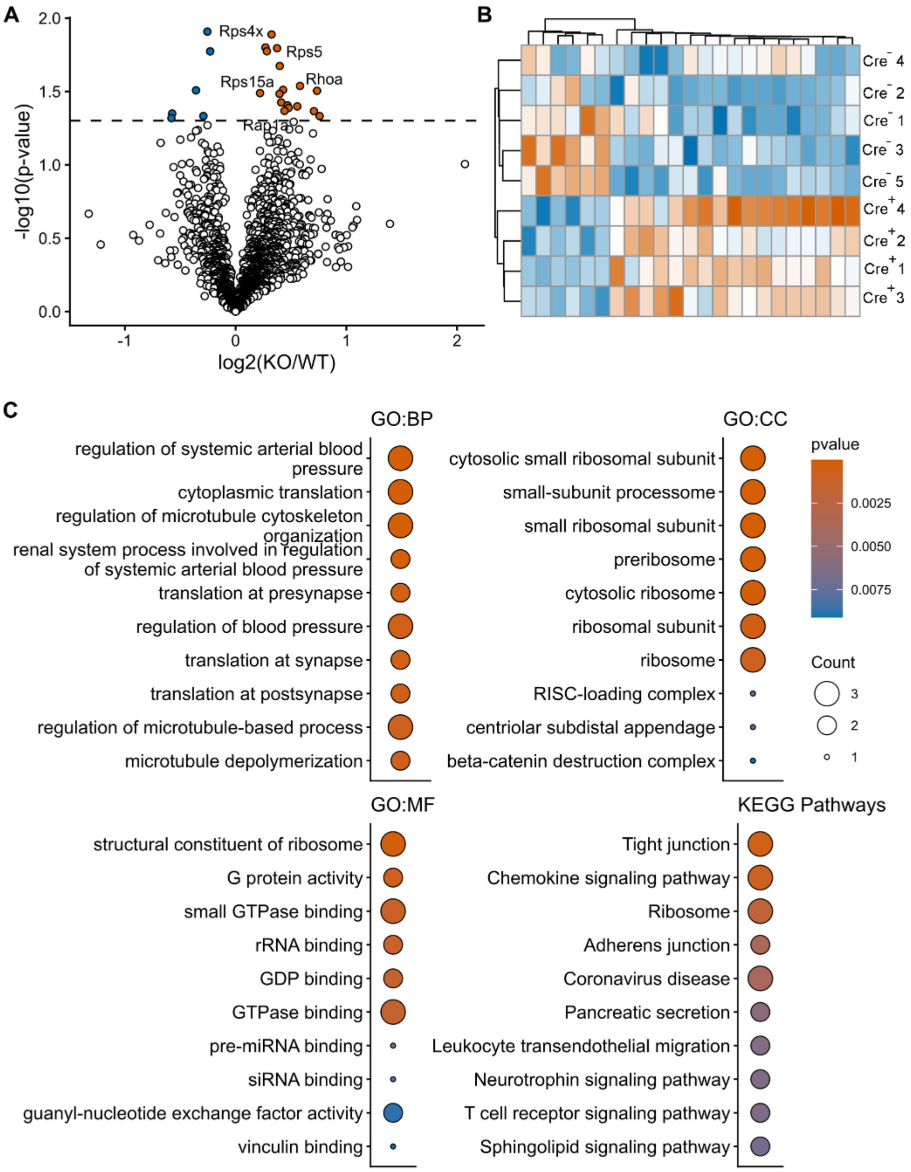

Figure S5. Differential protein abundance and functional enrichment in the unmodified proteome of Hrs-depleted post-synaptic membranes from mouse forebrain (cortex and hippocampus). (A) Volcano plot showing differentially abundant proteins in *Hrs*$^{f/f}$ *Syn1-Cre*$^{+/-}$ and *Hrs*$^{f/f}$*Syn1-Cre*$^{-/-}$ post-synaptic density (PSD2 fraction). The dashed horizontal line indicates the significance threshold at –log10 (0.05). A total of 2,064 proteins were identified (including splice variants), with 23 showing significant changes (17 increased and 6 decreased; $p < 0.05$). (B) Heatmap representing relative abundance of differentially expressed proteins across samples, hierarchically clustered. Color gradient indicates intensity of scaled relative peptide abundance (vermilion = high, blue = low). Sample labels correspond to clustering order (Cre^{-}1 to Cre^{-}5 and

bioRxiv preprint doi: https://doi.org/10.64898/2026.06.19.733273; this version posted June 24, 2026. The copyright holder for this preprint (which was not certified by peer review) is the author/funder, who has granted bioRxiv a license to display the preprint in perpetuity. It is made available under a CC-BY 4.0 International license.

$Cre^{+}1$ to $Cre^{+}4$). (C) Gene ontology (GO) classified gene sets show top biological process (BP), cellular component (CC), and molecular function (MF). KEGG enrichment analyses are also shown. The color gradient indicates unadjusted *p*-values. Point size corresponds to the number of genes enriched within each ontology or pathway.

bioRxiv preprint doi: https://doi.org/10.64898/2026.06.19.733273; this version posted June 24, 2026. The copyright holder for this preprint (which was not certified by peer review) is the author/funder, who has granted bioRxiv a license to display the preprint in perpetuity. It is made available under a CC-BY 4.0 International license.

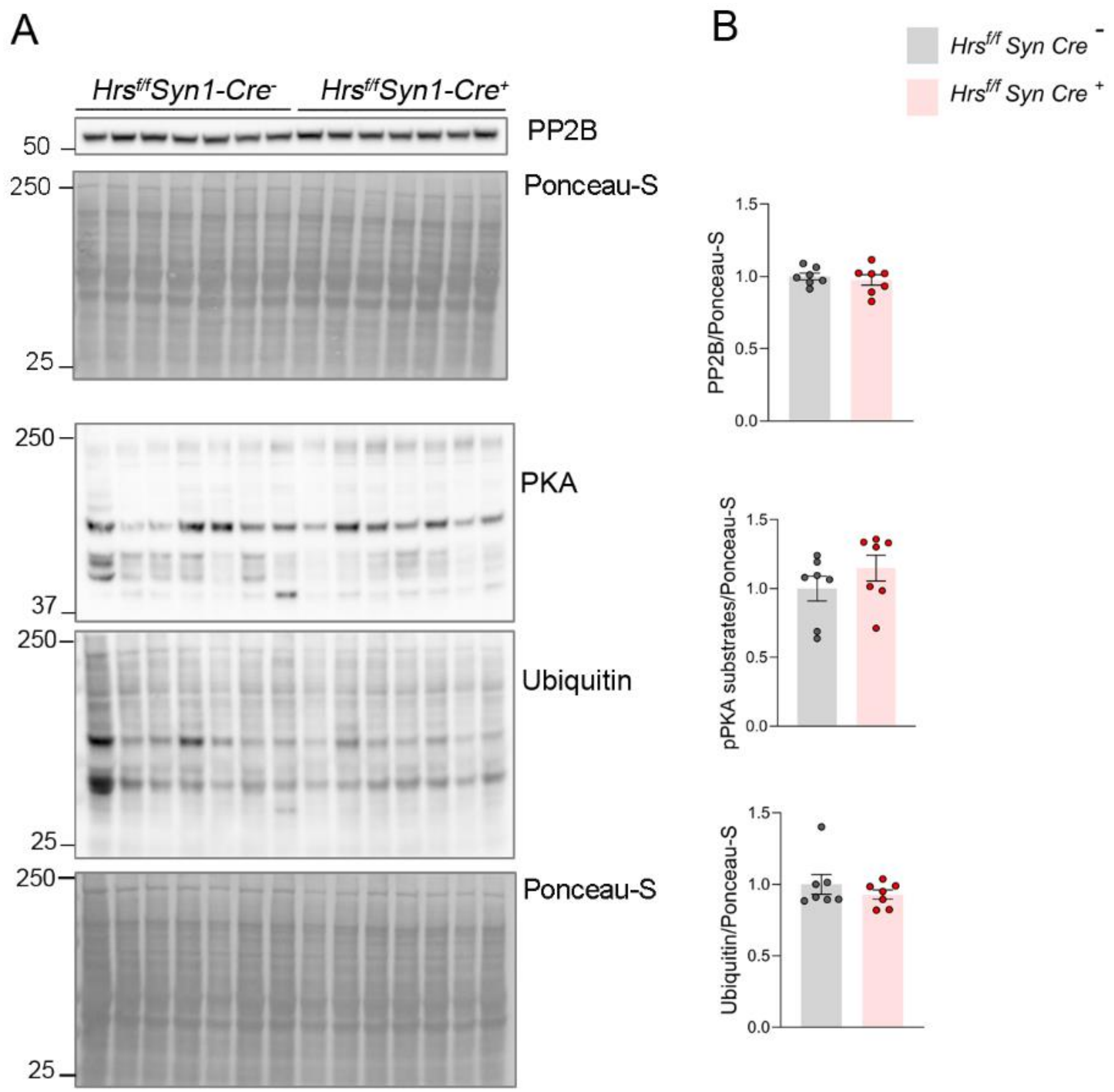

1114

1115 **Figure S6. Neuronal Hrs depletion in mice did not impact the phosphorylated PKA**
1116 **substrate, PP2B, or ubiquitin levels.** (A) Western blots of cortical lysates from *Hrs*$^{f/f}$*Syn1-Cre*$^{-/-}$
1117 and *Hrs*$^{f/f}$*Syn1-Cre*$^{+/-}$ mice for PKA substrates, PP2B, and ubiquitin (n = 7 per group). (B)
1118 Densitometric quantification of western blot signals. Mean ± SEM. Unpaired two-tailed *t*-test.

1119

bioRxiv preprint doi: https://doi.org/10.64898/2026.06.19.733273; this version posted June 24, 2026. The copyright holder for this preprint (which was not certified by peer review) is the author/funder, who has granted bioRxiv a license to display the preprint in perpetuity. It is made available under a CC-BY 4.0 International license.

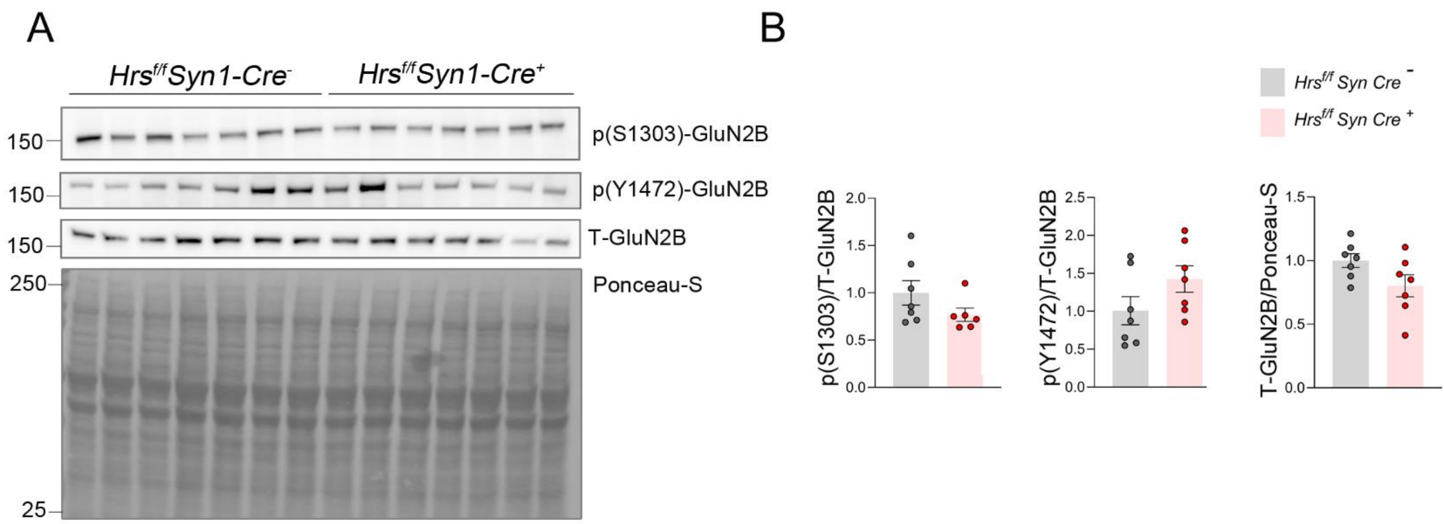

Figure S7. Neuronal Hrs depletion did not change the phosphorylation status of NMDA receptors. (A) Western blots of cortical lysates from *Hrs*$^{f/f}$*Syn1-Cre*$^{-/-}$ and *Hrs*$^{f/f}$*Syn1-Cre*$^{+/-}$ mice for GluN2B receptor and phosphorylation at S1303 and Y1472 (n = 7 per group). (B) Densitometric quantification of western blot signals. Mean ± SEM. Unpaired two-tailed Student's *t*-test.

bioRxiv preprint doi: https://doi.org/10.64898/2026.06.19.733273; this version posted June 24, 2026. The copyright holder for this preprint (which was not certified by peer review) is the author/funder, who has granted bioRxiv a license to display the preprint in perpetuity. It is made available under a CC-BY 4.0 International license.

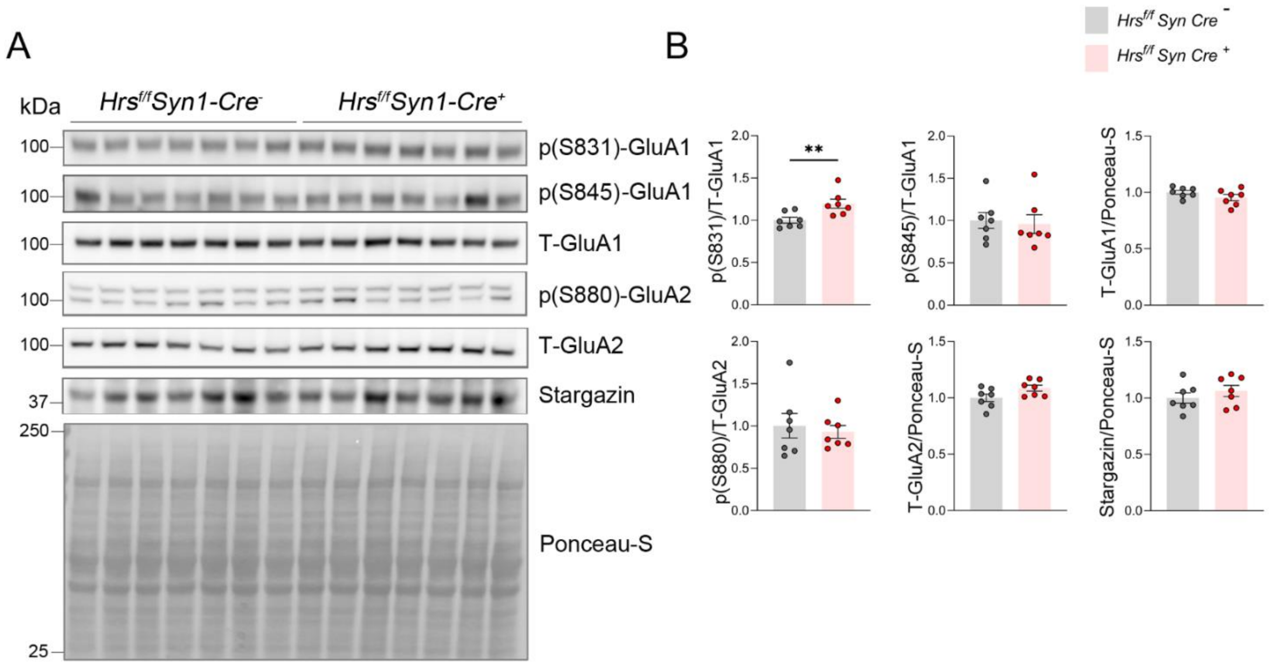

Figure S8. Neuronal Hrs depletion modestly enhances the phosphorylation of AMPAR subunit GluA1 at S831. (A) Western blots of cortical lysates from *Hrs*$^{f/f}$*Syn1-Cre*$^{-/-}$ and *Hrs*$^{f/f}$*Syn1-Cre*$^{+/-}$ mice for GluA1 and GluA2 subunits and their phosphorylation sites (n = 7 per group). (B) Densitometric quantification of western blot signals. Mean ± SEM. Unpaired two-tailed Student's *t*-test; ** $p < 0.01$.

bioRxiv preprint doi: https://doi.org/10.64898/2026.06.19.733273; this version posted June 24, 2026. The copyright holder for this preprint (which was not certified by peer review) is the author/funder, who has granted bioRxiv a license to display the preprint in perpetuity. It is made available under a CC-BY 4.0 International license.

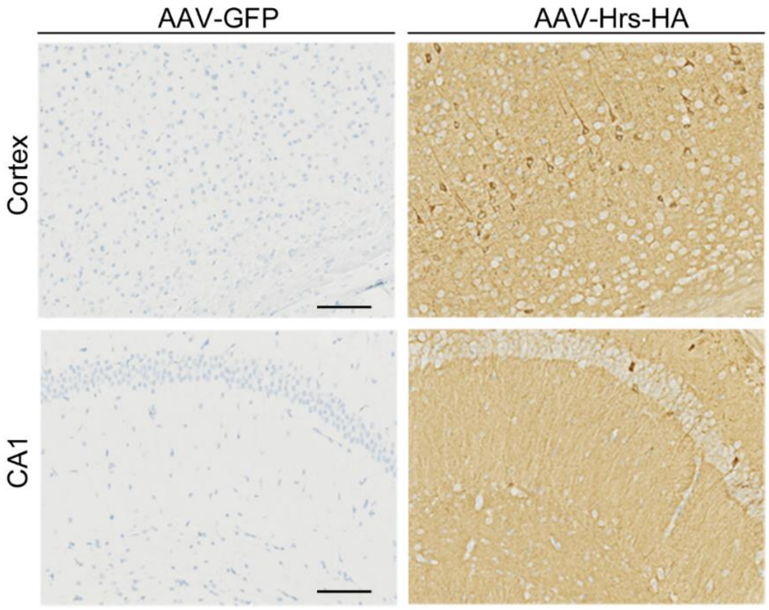

Figure S9. Robust neuronal expression of Hrs-HA in the cortex and hippocampus following AAV-Syn1-Hrs-HA transduction. Immunohistochemical detection of Hrs-HA in cortex and hippocampus (CA1) of adult WT mice three weeks after intravenous injection of AAV-Hrs-HA or control AAV-GFP (both of which express their transgene under the hSyn1 promoter). Strong HA immunoreactivity confirms robust neuronal Hrs overexpression. Scale bar = 100 µm.

bioRxiv preprint doi: https://doi.org/10.64898/2026.06.19.733273; this version posted June 24, 2026. The copyright holder for this preprint (which was not certified by peer review) is the author/funder, who has granted bioRxiv a license to display the preprint in perpetuity. It is made available under a CC-BY 4.0 International license.

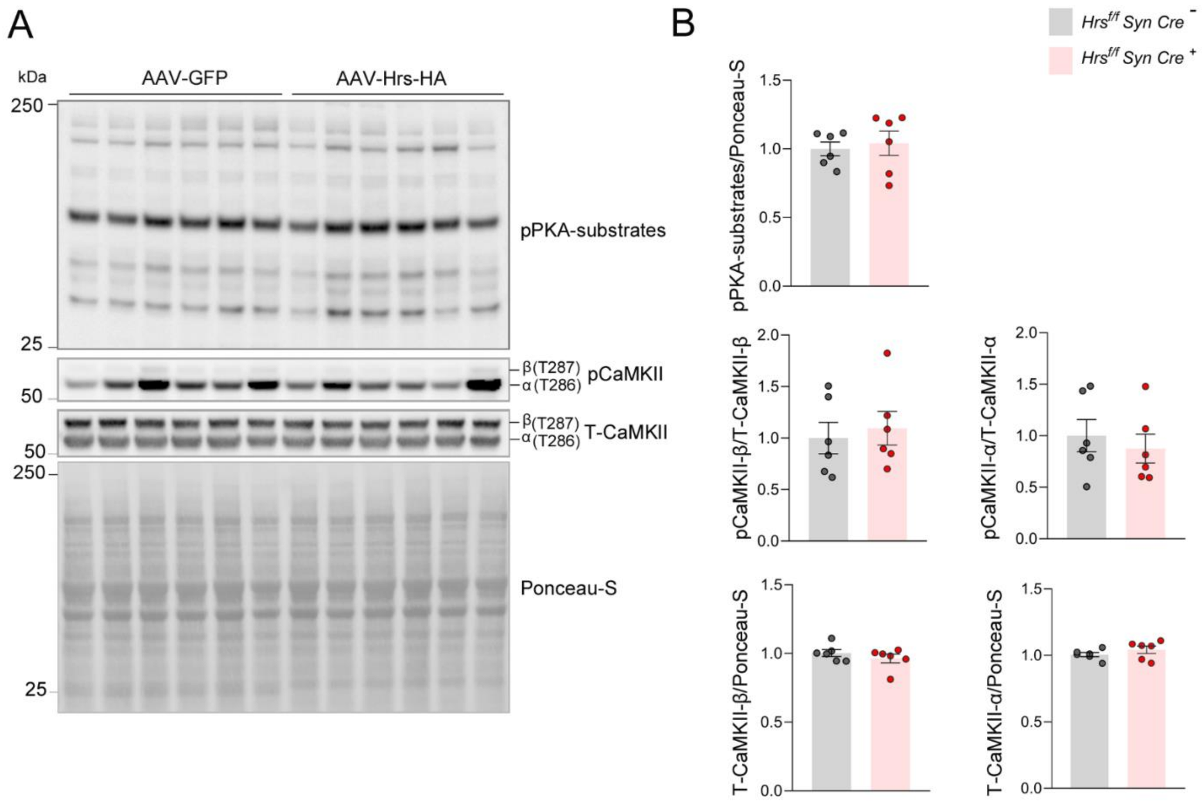

Figure S10. Neuronal Hrs overexpression in AAV-transduced mice did not affect the levels of phosphorylated or total CaMKII or phosphorylated PKA substrates. (A) Western blots of cortical lysates from adult WT mice injected with AAV-GFP or AAV-Hrs-HA (n = 6 per group), showing levels of phosphorylated PKA substrates (pPKA substrates) and phosphorylated and total CaMKII (p-T286) proteins. (B) Quantification of protein levels. Mean ± SEM. Unpaired two-tailed t-test.
